# Integrity of Newton’s cooling law based on thermal convection theory of heat transfer and entropy transfer

**DOI:** 10.1038/s41598-022-18961-8

**Published:** 2022-09-29

**Authors:** Bo Zhao

**Affiliations:** 1 School of Mechanical Engineering, Sichuan University, Chengdu, 610065 China; 2 State Key Laboratory of Mining Equipment and Intelligent Manufacturing, Taiyuan Heavy Machinery Group Co, Ltd, Taiyuan, 030024 China

**Keywords:** Phase transitions and critical phenomena, Thermodynamics

## Abstract

Although thermal convection is omnipresent in nature and technology and serves important purposes in various energy transport systems, whether convection can be viewed as an independent heat transfer means has long been argued The constant coefficient in the original version or convective heat transfer coefficient defined in the modern version of Newton’s cooling law quantifies the ratio of the surface heat flux to the temperature difference between a body surface and an adjacent fluid. However, none of the consistent analytical expressions for these two coefficients are present in Newton’s cooling law. The inherently complex relationship between these pending coefficients and convective heat flux vectors makes revealing the convective mechanism extremely difficult. Theoretical determination of these coefficients would bring new insights to thermal convection and direct applications to thermal management. Here we theoretically show consistent analytical expressions for the constant and convective heat transfer coefficients for various flows to make Newton’s cooling law a complete scientific law. For this purpose, a three-dimensional (3D) energy transfer theory of thermal convection is developed, and the convective heat flux vector, entropy flux vector and entropy generation rate inside the system are derived for both single-phase and phase-change flows. By recasting a control volume system into an equivalent control mass system and employing the first and second laws of thermodynamics, the fundamental advective heat transfer mode characterized by temperature differences and entropy changes is demonstrated. The physical implications underlying the 3D convective formulae are elucidated. Comparisons of the analytical results with laminar experiments and turbulent flow measurement benchmark data validate our theoretical findings. Our 3D heat and entropy transfer theory will broaden the research area of thermal convection processes and open up a new arena for the design and thermal management of convective heat transfer in single-phase and phase-change flows.

## Introduction

Thermal convection is universal phenomena in nature^1–10^, and a basic law of convective heat flux underlies the design, calculation and optimization of any convection heat transfer process^11–17^, which should render the unambiguous property relationship between a heat flux vector and its thermal driving force^18–26^. The total convective heat flux (i.e., the thermal energy flow per unit time and unit area^27^) is the superposition of two single heat transfer modes: advection due to macroscopic motion of a fluid and conduction due to random molecular motion (hereafter, *convection* refers to this cumulative transport, and *advection* refers to transport due to bulk fluid motion)^25–31^. In 1701, Newton described his cooling law for convective (or advective) heat transfer as follows^21–24^: *the rate of cooling of a warm body at any moment is proportional to the temperature difference between the body and its ambient fluid* ($- C/A{{{\text{d}}T_{s} } \mathord{\left/ {\vphantom {{{\text{d}}T_{s} } {{\text{d}}\tau }}} \right. \kern-\nulldelimiterspace} {{\text{d}}\tau }} = \alpha (T_{s} - T_{\infty } )$, where *C*, *A* and *T*_s_ are the heat capacity, surface area and temperature of the body, respectively, *T*_∞_ and *τ* are the constant fluid temperature and time, respectively, and the proportional coefficient *α* is called the advective constant and is associated with the fluid properties only). Unlike the original version, the modern version of Newton’s cooling law was incorporated by Fourier^22,32,33^ as the convective boundary condition on the wall surface ($- k\left. {{{\partial T} \mathord{\left/ {\vphantom {{\partial T} {\partial x_{2} }}} \right. \kern-\nulldelimiterspace} {\partial x_{2} }}} \right|_{{x_{2} = 0}} = h(T_{s} - T_{\infty } )$, where *k* is the thermal conductivity of the fluid, and *h* is defined as the convective heat transfer coefficient). Unlike *α*, *h* is not a property of the fluid, and its magnitude depends on all the variables that may influence the convective heat transfer process^26,28,34–37^. However, neither of the magnitudes of *α* and *h* is present in Newton’s law of cooling. Developing analytical expressions for *h* and *α* has always been a central focus and a difficulty of convective heat transfer problems. This development will eventually depend on the unclear energy transfer mechanism of thermal convection yet to be revealed. Some scientists regard convection as “conduction enhanced by fluid motion^15–18^” or as a means for “internal energy transport^20^” since the “convection” of energy owing to mass flow is not directly driven by a temperature difference^16,19,38^. Additionally, due to its inherent complexity, a unified heat transfer theory for general thermal convection has not been established thus far. Many attempts have been made to propose various convective heat flux theories^4–6,18,25,27,29–31,36,39–41^; however, analytical determination of the convective heat transfer coefficient and the advective constant based on the advective heat flux vector in single-phase and phase-change flows^13,14,42–45^ has rarely been explored.

Here we theoretically show consistent analytical expressions for the advective constant and convective heat transfer coefficients determined from the proposed general 3D energy transfer theory of thermal convection. By recasting a control volume system into an equivalent control mass system and employing the first and second laws of thermodynamics, an independent advection heat transfer mode characterized by temperature differences and entropy changes is demonstrated. The convective heat flux vector, entropy flux vector and entropy generation rate inside the system are derived for both single-phase and phase-change flows. The physical mechanism of thermal convection underlying these formulae is elucidated and clarified. The validity of the convective heat flux formulae is validated by comparison with laminar experiments and turbulent flow benchmark measurements. Our analysis reveals a clear physical implication of advective heat transfer due to bulk fluid motion and provides a unified theoretical approach to calculate the convective heat transfer coefficients for single-phase and phase-change flows.

## 3D energy transfer theory of thermal convection

### Independent advection heat transfer mode

**Figure 1:**
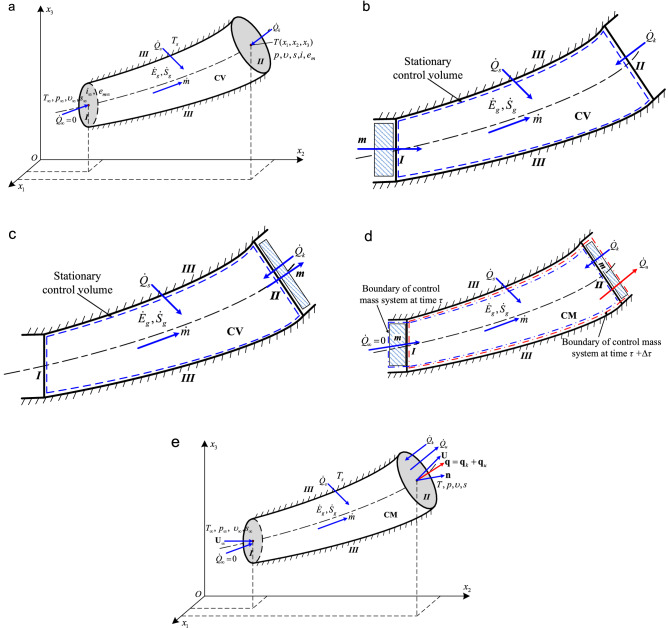
Advective heat transfer between two surfaces induced by mass flow. (**a**) Energy transfer due to mass flow in a Cartesian coordinate system. (**b**) CV system for a steady, compressible flow in a stationary tube with one inlet and one outlet, and its energy transfer at time *τ* and (**c**) at time *τ* + Δ*τ*. (**d**) Equivalent CM system recast from the above CV system for a steady compressible flow and its heat transfer at time *τ* and *τ* + Δ*τ*. (**e**) Heat transfer rates and heat flux vector at any surface *II* across which advection and conduction occur for the CM system, where **U**_∞_ is the constant velocity vector within the inlet section.

A steady flow of a single-phase, isotropic, compressible Newtonian fluid in a tube is considered. The control volume (CV) system, as shown in Fig. 1a, is enclosed by tube inlet section *I*, arbitrary section *II* within the fluid stream along the tube, and rigid wall surface *III* between *I* and *II*. The fluid moves at constant mass flow rate $\dot{m}$, where *m* is the mass entering (leaving) the CV, *i*_∞_, *e*_*m*∞_, *T*_∞_, *p*_∞_, *υ*_∞_, and *s*_∞_ represent the constant uniform specific enthalpy, specific mechanical energy, temperature, pressure, specific volume, and specific entropy maintained at inlet section *I*, respectively; *i*, *e*_*m*_, *T*, *p*, *υ*, and *s* are the specific enthalpy, specific mechanical energy, temperature, pressure, specific volume, and specific entropy at section *II*, respectively. At some initial time *τ,* the control mass (CM) system (Fig. 1d,e) is the sum of the mass within the CV at that instant and the mass *m* adjacent to inlet section *I*. At time *τ* + Δ*τ* this CM has moved such that all the mass originally in the region adjacent to the inlet is now just inside the CV. In the same time interval, part of the CM (equal to *m* for steady flow) has been pushed out of the CV into the region adjacent to section *II*. The mass flow rate $\dot{m} = \int_{S} {\rho {\mathbf{U}} \cdot {\mathbf{n}}} {\text{d}}S$, where *ρ* is the fluid density, ${\mathbf{U}} = \left\{ {u_{1} ,u_{2} ,u_{3} } \right\}$ designates the velocity vector at section *II*, and **n** is the unit vector pointing outward, normal to the cross-sectional area *A* of surface *II.* No shaft work and thermal radiation occur. All the parameters, including temperature, are considered to be uniform and constant within the inlet section (imagining that there exists an exterior heat source maintaining constant temperature *T*_∞_ at the inlet for the continuous flowing fluid). Additionally, the temperature gradient in any direction is assumed to be zero so that no thermal conduction occurs, hence the heat transfer rate $\dot{Q}_{\infty }$ at the inlet surface is zero. Assuming local thermodynamic equilibrium^19,44,46^ and applying the first and second laws of thermodynamics^38,46–50^ to the control volume (CV) system (Fig. 1a–c) and its equivalent control mass (CM) system (Fig. 1d,e), respectively, it gives

1 $$\dot{Q}_{s} + \dot{Q}_{k} + \dot{Q}_{\infty } + \dot{E}_{g} = \dot{m}\left[ {(i + e_{m} ) - (i_{\infty } + e_{m\infty } )} \right], \, \dot{S}_{g} + \dot{m}(s_{\infty } - s) + \dot{Q}_{s} /T_{s} + \dot{Q}_{k} /T + \dot{Q}_{\infty } /T_{\infty } = 0$$

2 $$\dot{Q}_{s} + \dot{Q}_{k} + \dot{Q}_{\infty } + \dot{E}_{g} = \dot{Q}_{u} , \, \dot{S}_{g} - {{\dot{Q}_{u} } \mathord{\left/ {\vphantom {{\dot{Q}_{u} } T}} \right. \kern-\nulldelimiterspace} T} + {{\dot{Q}_{s} } \mathord{\left/ {\vphantom {{\dot{Q}_{s} } {T_{s} }}} \right. \kern-\nulldelimiterspace} {T_{s} }} + {{\dot{Q}_{k} } \mathord{\left/ {\vphantom {{\dot{Q}_{k} } T}} \right. \kern-\nulldelimiterspace} T} + {{\dot{Q}_{\infty } } \mathord{\left/ {\vphantom {{\dot{Q}_{\infty } } {T_{\infty } }}} \right. \kern-\nulldelimiterspace} {T_{\infty } }} = 0$$

where $\dot{Q}_{s}$ and $\dot{Q}_{k}$ are the conductive heat transfer rates across rigid wall surface *III* and arbitrary flow surface *II*, respectively, $\dot{E}_{g}$, $\dot{S}_{g}$, and *T*_*s*_ are the thermal energy generation rate, entropy generation rate inside the system, and wall surface temperature, respectively, and $\dot{Q}_{u}$ is defined as the rate of energy transfer leaving across surface *II* for the CM system. Combining Eqs. (1) and (2) yields

3 $$\dot{Q}_{u} = \dot{m}\left[ {(i + e_{m} ) - (i_{\infty } + e_{m\infty } )} \right] = \dot{m}T(s - s_{\infty } )$$

The above equation not only indicates that the energy transfer rate $\dot{Q}_{u}$ which is defined only in the CM system (Fig. 1d,e), is the *net transfer rate of total energy* (specific total energy *e*_*t*_ = *i* + *e*_*m*_) between surface *II* and the inlet surface with different temperatures via mass flow, but also implies that $\dot{Q}_{u}$ equals the product of the fluid temperature and the change in entropy transfer. Energy can cross the boundary of a CM system only in the form of heat or work transfer^16,17,20,28,46–48^. It is demonstrated from Eq. (3) that the *net* total energy transfer by mass ($\dot{m}(e_{t} - e_{t\infty } )$, where $e_{t\infty } = i_{\infty } + e_{m\infty }$) is driven by the temperature difference^25^ and is accompanied by entropy transfer; according to the thermodynamic definition of heat transfer^16,38^, the energy transfer rate $\dot{Q}_{u}$ should be identified as heat transfer rather than work transfer. Note that $\dot{Q}_{u}$ equals the *net* total energy transfer rate between the arbitrary surface and the inlet surface due to bulk fluid motion (Fig. 1d,e). Therefore, $\dot{Q}_{u}$ is referred to as the *advective heat transfer rate*, and *Q*_*u*_ is accordingly called the *advective heat transfer*, which represents the net exchange of total energy by mass *m* between sections *II* and *I* with different temperatures, as if the common CV region were stationary (see Fig. 1d).

From Eqs. (1)–(3), the net specific entropy transfer *s* − *s*_∞_ due to the advective heat transfer rate $\dot{Q}_{u}$ and the entropy generation rate $\dot{S}_{g}$ within the system can be derived as

4 $$s - s_{\infty } = {{\left[ {(i + e_{m} ) - (i_{\infty } + e_{m\infty } )} \right]} \mathord{\left/ {\vphantom {{\left[ {(i + e_{m} ) - (i_{\infty } + e_{m\infty } )} \right]} T}} \right. \kern-\nulldelimiterspace} T}, \, \dot{S}_{g} = {{\dot{E}_{g} } \mathord{\left/ {\vphantom {{\dot{E}_{g} } {T_{s} }}} \right. \kern-\nulldelimiterspace} {T_{s} }} + (\dot{Q}_{u} - \dot{Q}_{k} )({1 \mathord{\left/ {\vphantom {1 T}} \right. \kern-\nulldelimiterspace} T} - {1 \mathord{\left/ {\vphantom {1 {T_{s} }}} \right. \kern-\nulldelimiterspace} {T_{s} }})$$

As derived above, heat advection is unambiguously distinguished from other energy transfer interactions, including mass flow and heat conduction. Based on this analysis, advective heat transfer can be considered a fundamental heat transfer mode in addition to conduction and radiation. This conclusion also clearly clarifies the previous controversy as to whether advection can be considered an independent heat transfer means. Notably, advective heat transfer occurs only if *net* energy transfer by mass occurs between *two* surfaces (such as sections *II* and *I* in Fig. 1d,e) with different temperatures in a fluid flow field. There cannot be any advective heat transfer across only *one* flow section where energy transport only occurs in the form of mass (for instance, at some arbitrary surface *II*, the *net* rate of energy transfer by mass $\dot{Q}_{u} = \dot{m}\left[ {(i + e_{m} ) - (i_{\infty } + e_{m\infty } )} \right]$, while the rate of energy transfer by mass is $\dot{m}(i + e_{m} )$). From an energy transfer perspective, advection is similar to *net radiation* between *two* surfaces rather than conduction across *one* surface.

### Convection in single-phase flows

Generally, the thermal energy being transferred includes the sensible energy in single-phase flows and the latent energy in phase-change flows^16,28,32^. We first examine the case of the convective heat flux vector and entropy flux vector in single-phase flows. Considering the Gibbs equations, Maxwell relation^27,47,48^, and advective heat transfer rate $\dot{Q}_{u} = \int_{S} {({\mathbf{q}}_{u} \cdot {\mathbf{n}}){\text{d}}S}$, the heat flux vector of advection **q**_*u*_ (W/m^2^) leaving across any surface *II* in a compressible flow is derived as^25^ (see Appendix)

5 $${\mathbf{q}}_{u} = \rho {\mathbf{U}}\left( {\int_{{T_{\infty } }}^{T} {c_{p} {\text{d}}T^{\prime}} - \int_{{p_{\infty } }}^{p} {{{\beta T} \mathord{\left/ {\vphantom {{\beta T} \rho }} \right. \kern-\nulldelimiterspace} \rho }{\text{d}}p^{\prime}} } \right), \, {\mathbf{q}}_{u} = \rho {\mathbf{U}}\left( {\int_{{T_{\infty } }}^{T} {c_{\upsilon } {\text{d}}T^{\prime}} + \int_{{\upsilon_{\infty } }}^{\upsilon } {{{\beta T} \mathord{\left/ {\vphantom {{\beta T} \kappa }} \right. \kern-\nulldelimiterspace} \kappa }{\text{d}}\upsilon^{\prime}} } \right)$$

where *β* is the volumetric coefficient of thermal expansion, *κ* is the isothermal compressibility, and *c*_*p*_ and *c*_*υ*_ are the specific heat capacity at constant pressure and constant volume, respectively. For 3D flow without internal heat source and viscous dissipation, we have demonstrated^25^ that an unsteady energy equation^27,36,52,57^ can be recast into $\frac{{{\text{d(}}\rho e_{t} )}}{{{\text{d}}\tau }} + \nabla \cdot {\mathbf{q}} = 0$, where *e*_*t*_ is the specific total energy and **q** is the total convective heat flux vector **q** = **q**_*u*_ + **q**_*k*_, with the conductive heat flux **q**_*k*_ determined by Fourier’s law^33^. We define the entropy flux vector **J**_*s*_ (W/(m^2^ K)) as the entropy flow per unit time and unit area crossing a surface^51^; thus, the entropy flux vector^47,49–51^ due to advection is ${\mathbf{J}}_{s} = {\mathbf{q}}_{u} /T$. From the foregoing relation $s - s_{\infty } = {{\left[ {(i + e_{m} ) - (i_{\infty } + e_{m\infty } )} \right]} \mathord{\left/ {\vphantom {{\left[ {(i + e_{m} ) - (i_{\infty } + e_{m\infty } )} \right]} T}} \right. \kern-\nulldelimiterspace} T}$ in Eq. (4), the net specific entropy transfer (*s* − *s*_∞_) due to advection is

6 $$s - s_{\infty } = {{\left( {\int_{{T_{\infty } }}^{T} {c_{p} {\text{d}}T^{\prime}} - \int_{{p_{\infty } }}^{p} {{{\beta T} \mathord{\left/ {\vphantom {{\beta T} \rho }} \right. \kern-\nulldelimiterspace} \rho }{\text{d}}p^{\prime}} } \right)} \mathord{\left/ {\vphantom {{\left( {\int_{{T_{\infty } }}^{T} {c_{p} {\text{d}}T^{\prime}} - \int_{{p_{\infty } }}^{p} {{{\beta T} \mathord{\left/ {\vphantom {{\beta T} \rho }} \right. \kern-\nulldelimiterspace} \rho }{\text{d}}p^{\prime}} } \right)} T}} \right. \kern-\nulldelimiterspace} T} = {{\left( {\int_{{T_{\infty } }}^{T} {c_{\upsilon } {\text{d}}T^{\prime}} + \int_{{\upsilon_{\infty } }}^{\upsilon } {{{\beta T} \mathord{\left/ {\vphantom {{\beta T} \kappa }} \right. \kern-\nulldelimiterspace} \kappa }{\text{d}}\upsilon^{\prime}} } \right)} \mathord{\left/ {\vphantom {{\left( {\int_{{T_{\infty } }}^{T} {c_{\upsilon } {\text{d}}T^{\prime}} + \int_{{\upsilon_{\infty } }}^{\upsilon } {{{\beta T} \mathord{\left/ {\vphantom {{\beta T} \kappa }} \right. \kern-\nulldelimiterspace} \kappa }{\text{d}}\upsilon^{\prime}} } \right)} T}} \right. \kern-\nulldelimiterspace} T}$$

If *c*_*p*_ and *c*_*υ*_ remain constant in Eq. (5), then ${\mathbf{q}}_{u} = \rho c_{p} {\mathbf{U}}\left( {T - T_{\infty } - \int_{{p_{\infty } }}^{p} {\frac{\beta T}{{\rho c_{p} }}{\text{d}}p^{\prime}} } \right)$ or ${\mathbf{q}}_{u} = \rho c_{\upsilon } {\mathbf{U}}\left( {T - T_{\infty } - \int_{{\upsilon_{\infty } }}^{\upsilon } {\frac{ - \beta T}{{\kappa c_{\upsilon } }}{\text{d}}\upsilon^{\prime}} } \right)$. The temperature changes caused by the density or dynamic pressure variations in a compressible flow are important for its heat balance^52^. By using some permissible simplifications^25,52^, the last term within the above parentheses represents the temperature difference in the adiabatic (isentropic) process caused by variations in pressure or density in the single-phase compressible flow^25^ (for example, the temperature increase due to adiabatic compression). Therefore, it must be deducted from the total temperature difference^52^ Δ*T* = *T* − *T*_∞_. For this purpose, we define this adiabatic temperature *T*_*ad*_ as the *potential temperature*^25^. Hence,

7 $${\mathbf{q}}_{u} = \rho c_{p} {\mathbf{U}}(T - T_{ad,p} ), \, {\mathbf{q}}_{u} = \rho c_{\upsilon } {\mathbf{U}}(T - T_{ad,\upsilon } ); \, s - s_{\infty } = c_{p} (1 - {{T_{ad,p} } \mathord{\left/ {\vphantom {{T_{ad,p} } T}} \right. \kern-\nulldelimiterspace} T}) = c_{\upsilon } (1 - {{T_{ad,\upsilon } } \mathord{\left/ {\vphantom {{T_{ad,\upsilon } } T}} \right. \kern-\nulldelimiterspace} T})$$

where ${\text{d}}T_{ad,p}^{{}} = \frac{{\beta T_{ad,p}^{{}} }}{{\rho c_{p} }}{\text{d}}p$, ${\text{d}}T_{ad,\upsilon }^{{}} = - \frac{{\beta T_{ad,\upsilon }^{{}} }}{{\kappa c_{\upsilon } }}{\text{d}}\upsilon$, and **q**_*u*_ has the same or opposite direction as **U**. Note that *T*_*ad*,*p*_ becomes the stagnation temperature^52^ when the velocity reduces to zero ($c_{p} (T_{ad,p} - T_{\infty } ) = \int_{{p_{\infty } }}^{p} {1/\rho {\text{d}}p} = (u_{\infty }^{2} - u^{2} )/2$ provided that *βT*_*ad,p*_ = 1, where *u*_∞_ is the free-stream velocity) in high-velocity compressible flows. Furthermore, the conductive heat flux **q**_*k*_ can be given from Fourier’s law^33^: ${\mathbf{q}}_{k} = - k\nabla T$. Therefore, for a single-phase, isotropic, compressible Newtonian fluid, the convective heat flux vector **q**(*x*_1_, *x*_2_, *x*_3_) = {*q*_1_, *q*_2_, *q*_3_} (Fig. 1d,e) is the resultant^25,27–29,39,40^ of the advective heat flux **q**_*u*_ and conductive heat flux **q**_*k*_:

8 $${\mathbf{q}} = {\mathbf{q}}_{u} + {\mathbf{q}}_{k} = \rho c_{p} {\mathbf{U}}(T - T_{ad,p} ) - k\nabla T,{\mathbf{q}} = {\mathbf{q}}_{u} + {\mathbf{q}}_{k} = \rho c_{\upsilon } {\mathbf{U}}(T - T_{ad,\upsilon } ) - k\nabla T$$

These two formulae for a compressible flow have the potential to be applied to actively cooled structures such as rocket engines^9,10^ and hypersonic vehicles under high aerodynamic thermal loads^8,53–56^.

We examine **q**_*u*_ in natural convection. Generally, the density *ρ* is a function of *p* and *T*, but the dependence of density on pressure can be ignored in flows that are affected by gravitation^52^. If *T* does not deviate too much from *T*_∞_, then use of the relation $\rho - \rho_{\infty } = - \beta \rho (T - T_{\infty } )$ is permissible^16,26,28,36^. Substituting this into ${\text{d}}T_{ad,\upsilon }^{{}} = - \frac{{\beta T_{ad,\upsilon }^{{}} }}{{\kappa c_{\upsilon } }}{\text{d}}\upsilon$ and integrating from *υ*_∞_ to *υ*, one obtains the difference in potential temperature as $T_{ad,\upsilon } - T_{\infty } = \frac{{\upsilon - \upsilon_{\infty } }}{{\kappa c_{\upsilon } }}\left[ {\frac{1}{2}(1 - \rho_{\infty } \upsilon ) - \beta T_{\infty } } \right]$. From Eq. (7), the advective heat flux vectors for free convection are obtained as follows

9 $${\mathbf{q}}_{u} = \rho c_{p} {\mathbf{U}}(T - T_{\infty } ) + \frac{{\rho {\mathbf{U}}\beta^{2} }}{{2\kappa \rho_{\infty } }}(T - T_{\infty } )^{2} , \, {\mathbf{q}}_{u} = \rho {\mathbf{U}}\left( {\frac{{\rho_{\infty } }}{\rho } - 1} \right)\left[ {\frac{{c_{p} }}{\beta } + \frac{1}{2\kappa }\left( {\frac{1}{\rho } - \frac{1}{{\rho_{\infty } }}} \right)} \right]$$

We now consider **q**_*u*_ for an incompressible flow. If the fluid velocity is not higher than one quarter the speed of sound, then the variations in pressure and specific volume can be neglected^36,57^, and the fluid can be treated as an incompressible medium. Hence, *c*_*p*_≈*c*_*υ*_ = *c*, *β* = 0, and the potential temperature *T*_*ad*_ degenerates into *T*_∞_^25^. If we let *θ* denote the temperature difference *θ* = *T* − *T*_∞_, then Eqs. (7) and (8) reduce to

10 $${\mathbf{q}}_{u} = \rho c{\mathbf{U}}\theta ; \, {\mathbf{q}} = {\mathbf{q}}_{u} + {\mathbf{q}}_{k} = \rho c{\mathbf{U}}\theta - k\nabla \theta ; \, s - s_{\infty } = c(1 - {{T_{\infty } } \mathord{\left/ {\vphantom {{T_{\infty } } T}} \right. \kern-\nulldelimiterspace} T})$$

From $\dot{S}_{g} = {{\dot{E}_{g} } \mathord{\left/ {\vphantom {{\dot{E}_{g} } {T_{s} }}} \right. \kern-\nulldelimiterspace} {T_{s} }} + (\dot{Q}_{u} - \dot{Q}_{k} )({1 \mathord{\left/ {\vphantom {1 T}} \right. \kern-\nulldelimiterspace} T} - {1 \mathord{\left/ {\vphantom {1 {T_{s} }}} \right. \kern-\nulldelimiterspace} {T_{s} }})$ in Eq. (4), the entropy generation rate inside the system becomes

11 $$\dot{S}_{g} = {{\dot{E}_{g} } \mathord{\left/ {\vphantom {{\dot{E}_{g} } {T_{s} }}} \right. \kern-\nulldelimiterspace} {T_{s} }} + ({1 \mathord{\left/ {\vphantom {1 T}} \right. \kern-\nulldelimiterspace} T} - {1 \mathord{\left/ {\vphantom {1 {T_{s} }}} \right. \kern-\nulldelimiterspace} {T_{s} }})\int_{S} {{\mathbf{q}} \cdot {\mathbf{n}}{\text{d}}S} = {{\dot{E}_{g} } \mathord{\left/ {\vphantom {{\dot{E}_{g} } {T_{s} }}} \right. \kern-\nulldelimiterspace} {T_{s} }} + ({1 \mathord{\left/ {\vphantom {1 T}} \right. \kern-\nulldelimiterspace} T} - {1 \mathord{\left/ {\vphantom {1 {T_{s} }}} \right. \kern-\nulldelimiterspace} {T_{s} }})\int_{S} {(\rho c{\mathbf{U}}\theta - k\nabla \theta ) \cdot {\mathbf{n}}{\text{d}}S}$$

For a 3D steady flow without internal heat source and viscous dissipation, its energy equation can be expressed by ∇∙**q** = ∇∙**q**_*u*_ + ∇∙**q**_*k*_ = 0, or **U**∙**q**_*k*_ − *a*∇∙**q**_*k*_ = 0, where ∇∙**q**_*u*_ =  − (**U**∙**q**_*k*_)/*a* and *a* = *k*/(*ρc*) is the thermal diffusivity. This implies that the divergence of **q**_*u*_ is thus *equivalent* to **q**_*k*_ enhanced by the velocity vector **U**, which well explains the foregoing argument on advection being “conduction enhanced by fluid motion”^15–19,58^. However, we have demonstrated that advection is an independent heat transfer mechanism that is completely different from conduction: advection is the net total energy transfer due to gross fluid movement, while conduction is the heat transfer due to random molecular motion.

In addition, Eq. (10) can be given in terms of its vectorial components in the cylindrical coordinate system^25^:

12 $$q_{r} = \rho cu_{r} \theta - k\frac{\partial \theta }{{\partial r}}, \, q_{\varphi } = \rho cu_{\varphi } \theta - \frac{k}{r}\frac{\partial \theta }{{\partial \varphi }}, \, q_{x} = \rho cu_{x} \theta - k\frac{\partial \theta }{{\partial x}}$$

where *q*_*r*_, *q*_*φ*_, and *q*_*x*_ are the heat flux components and *u*_*r*_, *u*_*φ*_, and *u*_*x*_ are the radial, circumferential and axial components of the velocity, respectively. Furthermore, in the turbulent flow of an incompressible fluid considering the fluctuations of velocity and temperature components, Eq. (10) can be recast in terms of heat flux components^30,31^:

13 $$q_{{x_{j} }} = \rho c\overline{u}_{j} (\overline{T} - T_{\infty } ){ + }\rho c\overline{{u^{\prime}_{j} T^{\prime}}} - k\frac{{\partial \overline{T}}}{{\partial x_{j} }}$$

where *j* = 1,2,3, $u^{\prime}_{j}$ is the velocity fluctuation term in the *x*_*j*_ direction, $T^{\prime}$ is the temperature fluctuation, and the overbar denotes the time-mean value; the detailed calculation of the fluctuation terms can be seen in references^30,31,59^ and^60^. We emphasize that Eqs. (10), (12) and (13) can also describe convective heat transfer (conduction plus advection) through a porous or permeable wall surface^31,55,56,61,62^.

### Convection in phase-change flows

**Figure 2:**
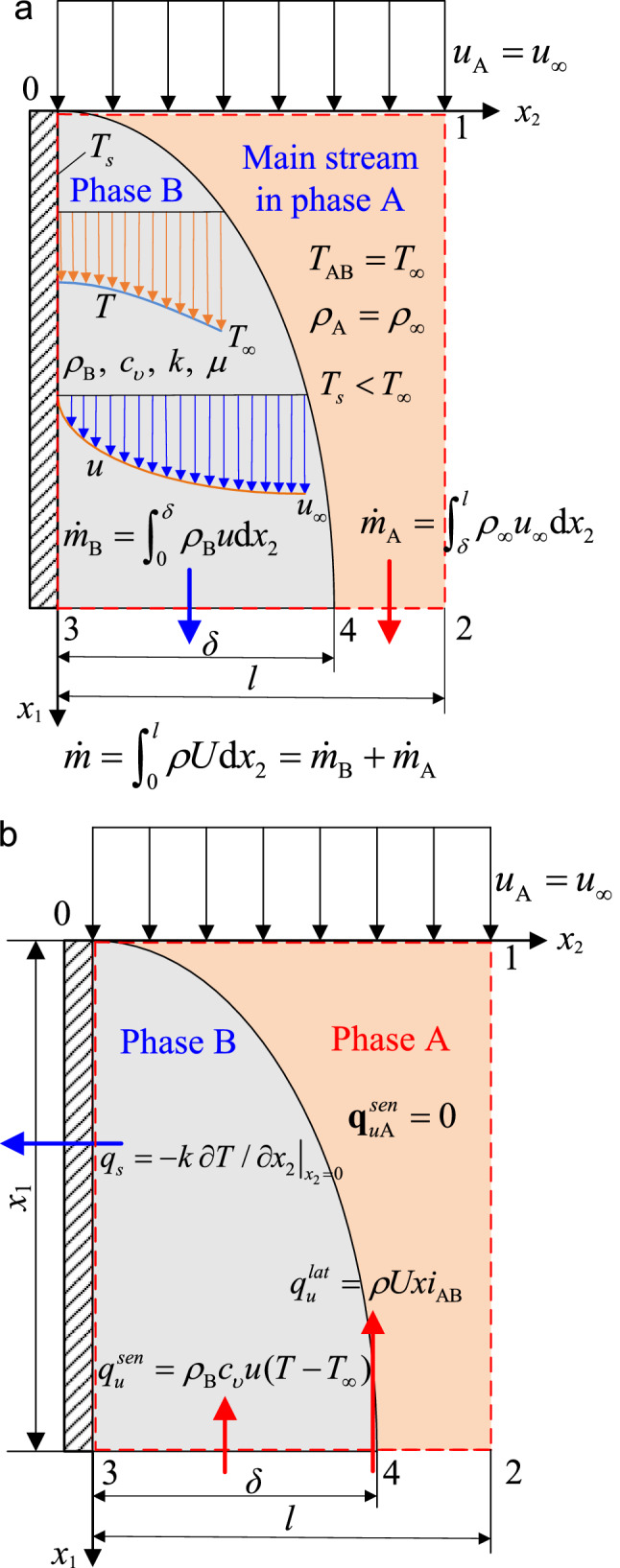
Convection in a phase-change flow (condensation). (**a**) Velocity and temperature profiles within the boundary-layer region of newly transitioned phase B (liquid). (**b**) Representation of the analytical heat flux vectors for the phase-change flow during forced convective processes. Here, $q_{s} = - k\left. {\partial T/\partial x_{2} } \right|_{{x_{2} = 0}}$ represents the wall surface heat flux due to conduction.

We now consider the case of the convective heat flux vector and entropy flux vector in phase-change flows. Condensation and evaporation are two important convective processes associated with the change in phase of a fluid in motion. Because there is a phase change, heat transfer to or from the fluid can occur without markedly influencing the fluid temperature (considering only first-order phase changes here; Fig. 2a). When a pure substance undergoes a phase transition from phase A to phase B at constant phase-change (or saturation) temperature *T*_AB_ (= *T*_∞_) and constant saturation pressure *p*_AB_, the specific volume *υ*, enthalpy *i*, and quality *x* of the two-phase mixture all change (*x* is the mass fraction of phase B; for the evaporation process, *x* is the vapor quality of the liquid–vapor mixture). However, only steady^63–65^ laminar phase-change flow is considered here for simplicity; hence, *υ*, *i* and *x* remain constant. When two phases coexist, the mixture is regarded as a pure compressible fluid, while each phase is taken as an incompressible fluid with constant properties. Mass transfer between the two phases is not considered, and a local equilibrium thermodynamic process is assumed^46,52^, in which every element can be considered a macroscopic thermodynamic subsystem^44^. Let *i*_AB_ be the specific latent heat of phase change (J/kg) that is equal to the specific enthalpy difference between the phase B and A fluids (*i*_AB_ = *i*_B_ − *i*_A_), representing the amount of energy needed to vaporize or condense a unit mass of the saturated phase at a given *T*_AB_ (or *T*_∞_). *υ*_A_ (*ρ*_A_, *i*_A_, *s*_A_, **U**_A_, **n**_A_, *S*_A_) and *υ*_B_ (*ρ*_B_, *i*_B_, *s*_B_, **U**_B_, **n**_B_, *S*_B_) denote the saturated specific volume (density, specific enthalpy, specific entropy, velocity vector, surface-normal unit vector, and surface area) of phases A and B, respectively. From the Clapeyron equation^27,47,48^, we have ${\text{d}}p/{\text{d}}T = (s_{{\text{B}}} - s_{{\text{A}}} )/(\upsilon_{{\text{B}}} - \upsilon_{{\text{A}}} ) = i_{{{\text{AB}}}} /[T_{\infty } (\upsilon_{{\text{B}}} - \upsilon_{{\text{A}}} )] = \beta /\kappa$, so *βT*_∞_/*κ* = *i*_AB_/(*υ*_B_ − *υ*_A_).

Similar to the specific heat capacity at constant pressure (or volume)^16,17,20,38,46–48^, the concept of the “*heat capacity at constant temperature*” *C*_*T*_ is introduced into the first-order phase-change process. *C*_*T*_ can be defined as *the specific enthalpy required to increase the volume of the unit mass of a substance by one cubic meter as the temperature is maintained constant in the phase-change process*. That is, *C*_*T*_ = d*i*/d*υ* or *C*_*T*_ = *i*_AB_/(*υ*_B_ − *υ*_A_) = *T*_∞_(*s* − *s*_A_)/(*υ* − *υ*_A_) = *βT*_∞_/*κ*, where *υ*≡1/*ρ* = (1 − *x*)*υ*_A_ + *xυ*_B_ and *s* = (1 − *x*)*s*_A_ + *xs*_B_ are the specific volume and specific entropy of the two-phase mixture with quality *x* = (*υ* − *υ*_A_)/(*υ*_B_ − *υ*_A_) = (*s* − *s*_A_)/(*s*_B_ − *s*_A_) = $\dot{m}_{{\text{B}}} /(\dot{m}_{{\text{A}}} + \dot{m}_{{\text{B}}} )$, where $\dot{m}_{{\text{A}}}$ and $\dot{m}_{{\text{B}}}$ are the mass flow rates of phases A and B (kg/s), respectively. Note that *C*_*T*_ is an intensive property parameter (Pa), and the mass flow rate of the two-phase mixture $\dot{m} = \dot{m}_{{\text{A}}} + \dot{m}_{{\text{B}}}$, i.e., $\int_{{S_{{\text{A}}} + S_{{\text{B}}} }} {\rho {\mathbf{U}} \cdot {\mathbf{n}}} {\text{d}}S = \int_{{S_{{\text{A}}} }} {\rho_{{\text{A}}} {\mathbf{U}}_{{\text{A}}} \cdot {\mathbf{n}}_{{\text{A}}} } {\text{d}}S_{{\text{A}}} + \int_{{S_{{\text{B}}} }} {\rho_{{\text{B}}} {\mathbf{U}}_{{\text{B}}} \cdot {\mathbf{n}}_{{\text{B}}} } {\text{d}}S_{{\text{B}}}$, remains constant during steady convective processes.

If *T* and *p* in Eq. (5) are chosen as state variables, then calculation of the advective heat flux for the first-order phase-change flow becomes impossible. Hence, we have to take *T* and *υ* as independent variables to determine **q**_*u*_. Provided that *c*_*υ*_ (or *βT*_∞_/*κ* = *C*_*T*_) is not a function of *T* (or *υ*) during the phase-change process, the advective heat flux vector in Eq. (5) becomes

14a $${\mathbf{q}}_{u} = \rho c_{\upsilon } {\mathbf{U}}(T - T_{\infty } ) + \rho C_{T} {\mathbf{U}}(\upsilon - \upsilon_{\infty } )$$

where *T*_∞_ = *T*_AB_ and *υ*_∞_ = *υ*_A_. The total advective heat flux for a phase-change flow consists of two contributions: the sensible energy transfer term ${\mathbf{q}}_{u}^{sen} = \rho c_{\upsilon } {\mathbf{U}}(T - T_{\infty } )$ due to the temperature difference and the latent energy transfer term ${\mathbf{q}}_{u}^{lat} = \rho C_{T} {\mathbf{U}}(\upsilon - \upsilon_{\infty } )$ due to the specific volume (or density) difference. Equation (14a) can also be rewritten by inserting the expression for *C*_*T*_:

14b $${\mathbf{q}}_{u} = \rho c_{\upsilon } {\mathbf{U}}\theta + \rho {\mathbf{U}}xi_{{{\text{AB}}}}$$

where $\theta = T - T_{\infty }$ is the temperature difference and *i*_AB_ = *i*_B_ − *i*_A_ becomes positive for evaporation and negative for condensation. Accordingly, the entropy flux vector **J**_*s*_ and the net specific entropy transfer *s* − *s*_A_ due to advection with a first-order phase change become

15 $${\mathbf{J}}_{s} = {{{\mathbf{q}}_{u} } \mathord{\left/ {\vphantom {{{\mathbf{q}}_{u} } T}} \right. \kern-\nulldelimiterspace} T} = \rho c_{\upsilon } {\mathbf{U}}(1 - T_{\infty } /T) + \rho {\mathbf{U}}xi_{{{\text{AB}}}} /T{; }s - s_{{\text{A}}} = c_{\upsilon } (1 - T_{\infty } /T) + xi_{{{\text{AB}}}} /T$$

Notably, the advective heat transfer in the first-order phase-change process is driven by the enthalpy difference *x*(*i*_B_ − *i*_A_) rather than the temperature difference *θ* as in single-phase flow, as indicated in Eq. (14b). Therefore, an appropriate definition of advective heat transfer for both single-phase and phase-change flows may be stated as follows: *advective heat transfer refers to the net total energy transfer between fluids with different temperatures or different phases by a macroscopic motion given by the fluid velocity vector, resulting from a spatial variation in enthalpy*.

Considering the phase-change convection process of condensation, as shown in Fig. 2a,b. Smooth laminar film condensation on a vertical, impermeable plate with unit width occurs in the forced convective process of a pure saturated vapor. The flow of phase A (vapor) maintains a constant velocity (*u*_A_ = *u*_∞_) and a constant density (*ρ*_A_ = *ρ*_∞_) at constant phase-change temperature *T*_∞_. Inlet section 01, section 32 at an arbitrary downstream distance from 01, solid wall 03, and section 12 in the main stream region constitute the CV system; *l* denotes the length of surface 32. Fluid motion is downward, and the vapor remains quiescent beyond outer edge 12. The vapor-side and interfacial resistances are negligible^63–65^; thus, the main contribution to the thermal resistance is from the liquid film (phase B). The interface temperature between phases A and B is assumed to be *T*_∞_. Here, *μ*, *k*, and *c*_*υ*_ are the dynamic viscosity (Pa s), thermal conductivity, and specific heat at constant volume of phase B (liquid), respectively, and *T*_*s*_, *δ*, *T*, and *u* are the constant wall surface temperature (lower than *T*_∞_), thickness, temperature and streamwise velocity of the liquid film, respectively. If thermal radiation, viscous dissipation, the mass transfer between the two phases, and the wall-normal component of velocity in the two-phase flow are ignored, then the heat advection associated with the sensible energy ${\mathbf{q}}_{u}^{sen}$ only occurs in the flow of newly transitioned phase B, such as in the boundary layer flow; however, the advection related to the latent energy ${\mathbf{q}}_{u}^{lat}$ occurs in the entire flow of phase A and phase B (Fig. 2b). From Eq. (14b), $q_{{u{\text{A}}}}^{sen} = 0$ due to zero temperature difference *θ* in the main stream (phase A), and $q_{{u{\text{B}}}}^{sen} = \rho_{{\text{B}}} c_{\upsilon } u\theta$ in the liquid film region (phase B), that is $$q_{u}^{sen} = \left\{ \begin{gathered} \rho_{{\text{B}}} c_{\upsilon } u\theta , \, 0 \le x_{2} \le \delta \hfill \\ 0, \, \delta \le x_{2} \le l \hfill \\ \end{gathered} \right.$$ and $$q_{u} = \left\{ \begin{gathered} \rho_{{\text{B}}} u(c_{\upsilon } \theta + xi_{{{\text{AB}}}} ), \, 0 \le x_{2} \le \delta \hfill \\ \rho_{\infty } u_{\infty } xi_{{{\text{AB}}}} , \, \delta \le x_{2} \le l \hfill \\ \end{gathered} \right.$$. These convective heat fluxes will provide a solid basis on the determination of convective heat transfer coefficients within single-phase and phase-change flows in the following.

## Heat transfer mechanism of thermal convection

### Advective constant in inviscid flows

**Figure 3:**
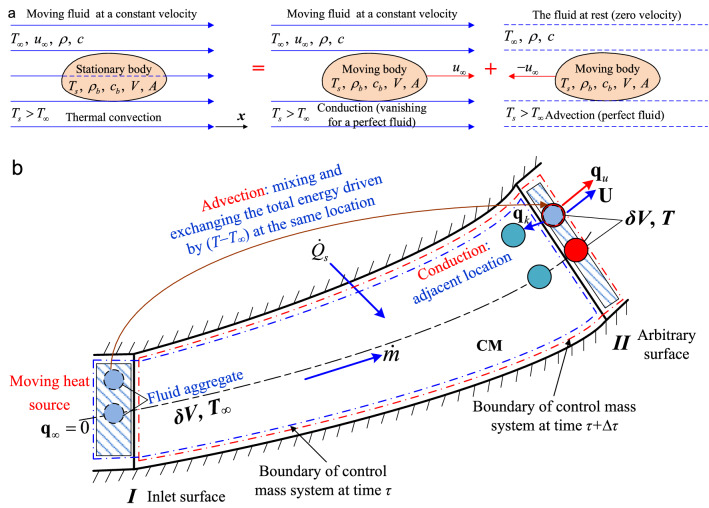
Heat transfer mechanism of thermal convection. (**a**) Original Newton’s law of cooling for an inviscid fluid with body transient temperature *T*_*s*_. (**b)** Advective and conductive heat transfer for a viscous fluid in steady flows.

We now show the mechanism of advective heat transfer considered in the original Newton’s law of cooling. A stationary, small-size, warm body with uniform temperature *T*_*s*_ is immersed in an extensive, incompressible, steady laminar flow with constant temperature *T*_∞_ and constant velocity *u*_∞_ (Fig. 3a). Here, *ρ*_*b*_, *c*_*b*_, *V*, and *A* are the density, specific heat, volume, and surface area of the body, respectively. Thermal radiation in the convective process is ignored. The physical size of the body, compared with that of the fluid stream, is assumed to be so small (resembling the cooling process of a cup of coffee in a room or isolated ships drifting in an ocean^24^) that its internal thermal resistance is negligible. Accordingly, the interior conduction within the body can be neglected, resulting in a *uniform* temperature distribution (*T*_*s*_) *throughout* the body^25,35^. Additionally, the original cooling law is valid only for a small temperature difference between the body and ambient fluid (*T*_*s*_ − *T*_∞_ = 20 ~ 30 K)^21–23^, so the exterior conduction in the fluid may also be ignored compared with the advection that occurs. Therefore, the flowing fluid considered in the cooling law might be simplified as a *perfect fluid* (inviscid fluid). We emphasize that the convective heat transfer scenario of a static body in a constant-velocity (*u*_∞_) and constant-temperature (*T*_∞_) fluid stream can be considered the superposition of two single heat transfer modes (Fig. 3a): the heat transfer induced by the body moving with the same velocity *u*_∞_ as the stream and that induced by the body moving in its own plane with constant velocity − *u*_∞_ in the opposite direction within the infinite, quiescent fluid (provided that the observer is located in the fluid stream). The former heat transfer is easily identified as the conductive heat transfer mode due to no relative macroscopic motion between the body and the fluid. However, this heat transfer subsides (**q**_*k*_ = 0) for a perfect fluid owing to the zero temperature gradient throughout the fluid flow field. Now, we shall demonstrate that the latter heat transfer mode can independently be considered advection. We consider the scenario in which a swimmer, with uniform body temperature *T*_*s*_ greater than ambient pool temperature *T*_∞_, is moving in their own plane with constant velocity − *u*_∞_ in a large, cold pool filled with water at constant temperature *T*_∞_ (Fig. 3a). During the same time interval Δ*τ*, the equal mass *m* of water displaced by the body carries away heat equal to *cm*(*T*_*s*_ − *T*_∞_) from the body due to advection. The size of the human body is relatively small compared with the swimming pool such that its cross-sectional area *S* can be approximated by the surface area *A*, hence, the mass flow rate $\dot{m}$ of water displaced by the body equals *ρu*_∞_*A*. Therefore, the advective heat transfer rate is expressed as *cρu*_∞_*A*(*T*_*s*_ − *T*_∞_). Compared with the original rate equation in Newton’s law of cooling $- c_{b} \rho_{b} V{{{\text{d}}T_{s} } \mathord{\left/ {\vphantom {{{\text{d}}T_{s} } {{\text{d}}\tau }}} \right. \kern-\nulldelimiterspace} {{\text{d}}\tau }} = \alpha A(T_{s} - T_{\infty } )$, the advective constant *α* is determined as $\alpha = \rho cu_{\infty }$. We define the changing temperature difference *θ*_*s*_ = *T*_*s*_ − *T*_∞_, and note that the magnitude of the advective heat flux vector becomes *q*_*u*_ = *ρcu*_∞_*θ*_*s*_, which is identical to Eq. (10). The advective heat loss from the body is evidenced as a decrease in the internal energy of the body; therefore, *αA*(*T*_*s*_ − *T*_∞_) =  − *c*_*b*_*ρ*_*b*_*V*d(*T*_*s*_ − *T*_∞_)/d*τ*, or d*θ*/*θ* =  − *ρcu*_∞_*A*/(*ρ*_*b*_*c*_*b*_*V*)d*τ*. From the initial condition *θ* = *θ*_0_ = *T*_0_ − *T*_∞_ (or *T*_*s*_ = *T*_0_) when *τ* = 0, we obtain $\theta = \theta_{0} e^{{ - \rho cu_{\infty } A/(\rho_{b} c_{b} V)\tau }}$. This represents the complete original version of Newton’s law of cooling^21–24^ with the determined advective constant *α*. Note that *α* is proportional to *u*_∞_, as has been experimentally validated by Newton^21,22^, Richmann^24^, Fourier^33^, and others^23^. Unlike the convective heat transfer coefficient *h*, *α* = *ρcu*_∞_ is a constant involving only the fluid properties, and the bridge between the two is the Stanton number: St = *h*/*α*. Special attention is given to the body surface heat flux **q**_*s*_ due to advection for a perfect fluid rather than conduction for a viscous fluid; hence, **q**_*s*_ should be equal to **q**_*u*_, whose direction is the same as *u*_∞_ instead of the wall-normal direction for *h* (Fig. 3a).

### Convective heat flux vector in viscous flows

If the size of a body or solid surface is not very small, then the viscous effect of the real fluid must be considered, and a thermal boundary layer will develop^52^. Hence, the convective process involves not only advection but also conduction. For simplicity, the mechanism of convective (advective plus conductive) heat transfer is discussed only for an incompressible viscous fluid in steady flows (Fig. 3b). From the derivation of Newton’s law of cooling (Fig. 3a), it is noted that the fluid in the ambient stream maintaining constant temperature *T*_∞_ is equivalent to a moving heat source^66^. Similarly, the free stream or fluid at the inlet surface (as well as its exterior surroundings) in an external or internal flow can also be regarded as a moving heat source (*T*_∞_) in the advective heat transfer process. Therefore, inlet surface *I* can be regarded as a moving heat source (no advection or conduction occurs due to zero temperature difference or gradient while both advective and conductive heat transfer modes are induced at other arbitrary surface *II*) in Fig. 3b. Such a movable heat source embedded in the fluid aggregate with the *incremental volume δV* is transported throughout the fluid flow field similar to a conveyor belt pushed by flow work. The fluid bulk motion is associated with the fact that at any instant, large numbers of molecules are moving collectively or as aggregates^28^. Hence, the concept of the *incremental volume δV* is introduced^46^, which is defined as *the smallest physical volume containing large numbers of particles in a fluid-flow field that is macroscopically large enough to be considered ****uniform**** in temperature and velocity throughout*. This fluid aggregate with incremental volume *δV* for a viscous fluid plays much the same role as the foregoing *small-size* body with volume *V* in the original Newton’s law of cooling for a perfect fluid.

During the same time interval Δ*τ*, at any flowing position (section *II* in Fig. 3b), an equal volume of fluid aggregate of temperature *T*, mixing and exchanging its total energy with this moving heat source *at the same location*, and displaced by the moving heat source aggregate with the incremental volume *δV*, carries with it the *net* total energy leaving this location with velocity vector **U** equal to *cρδV*(*T* − *T*_∞_), as shown in Fig. 3b, hence, the advective heat transfer rate becomes *cρ*(*T* − *T*_∞_)*δV*/Δ*τ*. Since $\mathop {\lim }\limits_{\Delta \tau \to 0} \delta V/\Delta \tau = {\mathbf{U}}S$ (all the parameters in *δV* are uniform, and *S* is the cross-sectional area of *δV*), according to the definition of heat flux^25,27^, the advective heat flux vector yields **q**_*u*_ = *ρc***U**(*T* − *T*_∞_), which is again identical to Eq. (10). This may be considered the energy transfer mechanism of advection indicated in Eq. (10). On the other hand, because of the motion of the fluid, the fluid aggregate leaving by advection simultaneously much more rapidly exchanges its heat by conduction with the adjacent fluid aggregates *at different locations* than if the same viscous medium were at rest (Fig. 3b). This also well explains the conduction heat transfer in the convective process^25,58^.

**Figure 4:**
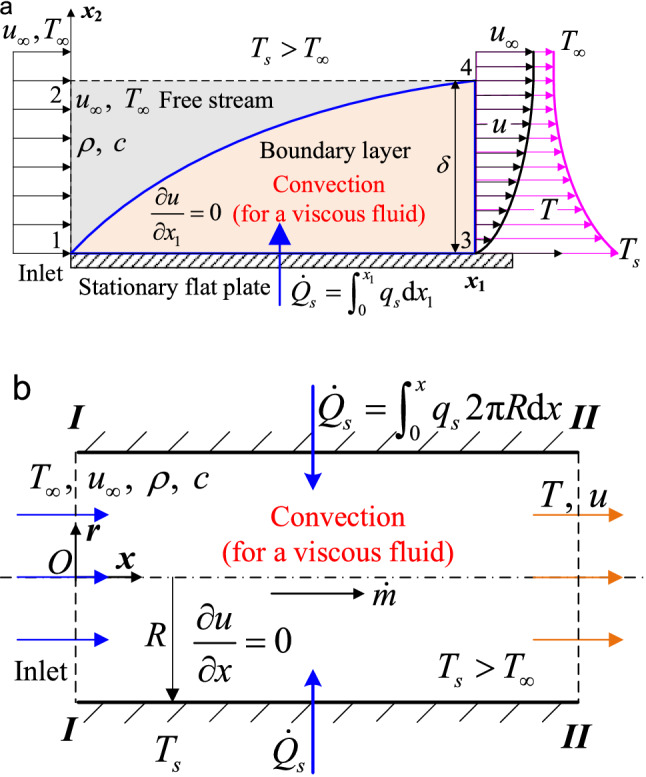
Determination of heat transfer coefficients based on the convective heat flux vector. (a**)** External flows. Here the hydrodynamic and thermal boundary layers have the same thickness *δ* and originate at *x*_1_ = 0. (**b**) Internal flows. A fluid with uniform and constant temperature *T*_∞_ enters a tube of radius *R* with uniform and constant velocity *u*_∞_ and constant mass flow rate $\dot{m}$ in a steady laminar flow.

To clearly show the energy transfer mechanism of advection, we emphasize that the inlet surface is considered the unique surface with $\dot{Q}_{\infty } = 0$, across which all the parameters, especially temperature, should be uniform and constant to guarantee the uniqueness of the advective heat flux in a steady flow^40^. Note that such an inlet surface of the free stream is evident in an external flow (see the following Fig. 4a). In emphasizing this requirement for an internal flow (Fig. 4b), however, it is implicitly assumed that the temperature is uniform across the inlet cross-sectional area, which is not true in reality if convective heat transfer occurs^28^. Therefore, the average temperature across the cross-sectional area of the inlet should be regarded as the uniform and constant inlet temperature *T*_∞_.

## Expression for convective heat transfer coefficients

### Two simplified formulae

Consider the two-dimensional (2D), steady, laminar, viscous, single-phase flow of an incompressible, constant-property fluid over an impermeable plate with unit width (inlet section 12, section 34 at an arbitrary downstream distance from 12, solid wall 13, and section 24 in the free-stream region constitute the CV system, as shown in Fig. 4a) or through an impermeable pipe (Fig. 4b). No internal heat source (e.g., radiation, chemical reactions, Joule heating) is generated, namely, $\dot{E}_{g} = 0$. The viscous dissipation and streamwise conduction are ignored. If ∂*u*/∂*x*_1_ (or ∂*u*/∂*x*) is equal to zero, then the wall-normal component of the velocity vanishes, *u*_∞_ is a constant, and *u* remains constant in the *x*_1_(*x*) direction. Here *T*_*s*_, *T*_∞_, and $\dot{Q}_{s}$ are the wall surface temperature, free-stream or inlet temperature, and conductive heat transfer rate of the fluid on the wall, respectively. According to the energy balance, the rate of conductive heat transfer entering across the impermeable wall is equal to the rate of advective heat transfer leaving across surface 34 in Fig. 4a (or section *II* in Fig. 4b). Following the convective heat flux Eq. (10) yields $\dot{Q}_{s} = \int_{0}^{{x_{1} }} {q_{s} {\text{d}}x_{1} } = \dot{Q}_{u} = \int_{0}^{\delta } {q_{u} {\text{d}}x_{2} } = \int_{0}^{\delta } {\rho cu\theta {\text{d}}x_{2} }$ for an external flow, and $- \int_{0}^{x} {q_{s} {2}\pi R{\text{d}}x} = \int_{0}^{R} {\rho cu\theta {2}\pi r{\text{d}}r}$ (the conductive heat flux *q*_*s*_ on the wall surface is negative in the cylindrical coordinate system plotted in Fig. 4b) for an internal flow. Differentiating both sides of each equation with respect to *x*_1_ (*x*) and considering the definition *q*_*s*_ = *h*(*T*_*s*_ − *T*_∞_) for an external flow and *q*_*s*_ = *h*(*T*_*av*_ − *T*_*s*_) for an internal flow (*T*_*av*_ is the average temperature of fluid across any cross-sectional area for an internal flow)^36,57,67^, we obtain

16 $$h = \frac{1}{{T_{s} - T_{\infty } }}\frac{{\text{d}}}{{{\text{d}}x_{1} }}\int_{0}^{\delta } {\rho cu\theta {\text{d}}x_{2} } {\text{ (external flow); }}h = \frac{R}{{T_{s} - T_{av} }}\frac{{\text{d}}}{{{\text{d}}x}}\int_{0}^{1} {\rho cu\theta \eta {\text{d}}\eta } {\text{ (internal flow)}}$$

where *η* = *r*/*R*. Equation (16) establishes the inherent energy transfer relationship between the wall-normal surface heat conduction and the streamwise heat advection bridging *h* and **q**_*u*_.

### General expression in incompressible external flows

Special attention is given to the 2D boundary layer laminar flow over a flat plate (Fig. 4a), whose energy equaion^26–28,34–36,52^ is $\rho c\left( {u_{1} \frac{\partial \theta }{{\partial x_{1} }} + u_{2} \frac{\partial \theta }{{\partial x_{2} }}} \right) = \frac{\partial }{{\partial x_{2} }}\left( {k\frac{\partial \theta }{{\partial x_{2} }}} \right)$. Integrating *x*_2_ from 0 to *δ* on both sides gives $\int_{0}^{\delta } {\nabla \cdot {\mathbf{q}}_{u} } {\text{d}}x_{2} = - \int_{0}^{\delta } {{{{\mathbf{U}} \cdot {\mathbf{q}}_{k} } \mathord{\left/ {\vphantom {{{\mathbf{U}} \cdot {\mathbf{q}}_{k} } a}} \right. \kern-\nulldelimiterspace} a}} {\text{d}}x_{2} = q_{s}$, where **q**_*u*_ and **q**_*k*_ are presented by Eq. (10). Under the boundary layer assumption condition, note that the convective heat transfer on the wall can be viewed as augmentation of conduction in a fluid by the velocity vector (i.e., advection)^18,56^. However, this does not imply that advection is not a fundamental mechanism of heat transfer. Following the definition of *h* and recalling $\int_{0}^{\delta } {\nabla \cdot {\mathbf{q}}_{u} } {\text{d}}x_{2} = q_{s}$ gives the general expression of *h* in incompressible external flows as follows

17a $$h = \frac{1}{{\theta_{s} }}\int_{0}^{\delta } {\nabla \cdot {\mathbf{q}}_{u} } {\text{d}}x_{2}$$

where *θ*_*s*_ = *T*_*s*_ − *T*_∞_. The components of velocity and advective heat flux normal to the wall subside provided that ∂*u*/∂*x*_1_ = 0. Thus, for an isothermal wall condition, Eq. (17a) reduces to $h = \frac{{\text{d}}}{{{\text{d}}x_{1} }}\int_{0}^{\delta } {\rho cu\theta /} \theta_{s} {\text{d}}x_{2}$, which is equivalent to the integral energy equation^19,26,34–36,57^. For a constant heat flux wall condition, $h = \frac{1}{{\theta_{s} }}\frac{{\text{d}}}{{{\text{d}}x_{1} }}\int_{0}^{\delta } {\rho cu\theta } {\text{d}}x_{2}$, and $h_{av} = \frac{1}{{\theta_{s} x_{1} }}\int_{0}^{\delta } {\rho cu\theta } {\text{d}}x_{2}$, where *h*_*av*_ is the average convective heat transfer coefficient^28^.

### General expression in incompressible internal flows

A 2D axially symmetric, steady laminar flow in a pipe is considered (Fig. 4b). The energy equation^26–28,34–36,52^ in cylindrical coordinates is $\rho c\left( {u\frac{\partial \theta }{{\partial x}} + u_{r} \frac{\partial \theta }{{\partial r}}} \right) = \frac{1}{r}\frac{\partial }{\partial r}\left( {rk\frac{\partial \theta }{{\partial r}}} \right) + \frac{\partial }{\partial x}\left( {k\frac{\partial \theta }{{\partial x}}} \right)$, where *u*_*r*_ is the wall-normal (radial) component of velocity. This equation can also be expressed in terms of its convective heat flux components in Eq. (12): $- r\frac{{\partial q_{x} }}{\partial x} = \frac{{\partial (rq_{r} )}}{\partial r}$. Integrating both sides with respect to *r* from 0 to *R* gives $- \int_{0}^{R} {\frac{r}{R}\frac{{\partial q_{x} }}{\partial x}} {\text{d}}r = q_{s}$; thus, the general expression of *h* in incompressible internal flows

17b $$h = \frac{1}{{T_{s} - T_{av} }}\int_{0}^{R} {\frac{r}{R}\frac{{\partial q_{x} }}{\partial x}} {\text{d}}r$$

If the axial conduction is ignored, then Eq. (17b) degenerates to $h = \frac{R}{{T_{s} - T_{av} }}\frac{{\text{d}}}{{{\text{d}}x}}\int_{0}^{1} {\rho cu\theta \eta } {\text{d}}\eta$, which is identical to Eq. (16). Considering the wall condition of constant heat flux *q*_*s*_ and the energy balance gives $2{\uppi }Rq_{s} x = c\dot{m}(T_{av} - T_{\infty } )$, i.e., $T_{av} = T_{\infty } + {{2{\uppi }Rq_{s} x} \mathord{\left/ {\vphantom {{2{\uppi }Rq_{s} x} {(c\dot{m}}}} \right. \kern-\nulldelimiterspace} {(c\dot{m}}})$, as shown in Fig. 4b.

### Film condensation on a vertical plate

Consider the 2D, steady, laminar, viscous, phase-change flow of a compressible, liquid–vapor mixture over an impermeable, smooth, vertical plate with unit width (Fig. 2a). No internal heat source is generated, and the viscous dissipation, radiation, streamwise conduction, and wall-normal advection are neglected. Since the rate of conductive heat transfer leaving across the impermeable wall is equal to the sum of the advective heat transfer rate associated with the sensible energy entering across surface 34 and that associated with the latent energy entering across surface 32 (Fig. 2b), it gives $\int_{0}^{{x_{1} }} {q_{s} {\text{d}}x_{1} } = \int_{0}^{\delta } {q_{u}^{sen} {\text{d}}x_{2} } + \int_{0}^{l} {q_{u}^{lat} {\text{d}}x_{2} }$; Inserting Eq. (14b) yields $\int_{0}^{{x_{1} }} {q_{s} {\text{d}}x_{1} } = \int_{0}^{\delta } {\rho_{{\text{B}}} c_{\upsilon } u(T - T_{\infty } ){\text{d}}x_{2} + } \int_{0}^{l} {\rho Uxi_{{{\text{AB}}}} {\text{d}}x_{2} } = \int_{0}^{\delta } {\rho_{{\text{B}}} c_{\upsilon } u(T - T_{\infty } ){\text{d}}x_{2} + } \dot{m}_{{\text{B}}} i_{{{\text{AB}}}}$. Differentiating both sides with respect to *x*_1_ and considering the definition *q*_*s*_ = *h*(*T*_*s*_ − *T*_∞_) for film condensation^63–65^, we obtain the convective heat transfer coefficient *h* for condensation on a vertical plate

18 $$h = \frac{1}{{T_{s} - T_{\infty } }}\left[ {\frac{{{\text{d}}\dot{m}_{{\text{B}}} }}{{{\text{d}}x_{1} }}i_{{{\text{AB}}}} + \frac{{\text{d}}}{{{\text{d}}x_{1} }}\int_{0}^{\delta } {\rho_{{\text{B}}} c_{\upsilon } u(T - T_{\infty } ){\text{d}}x_{2} } } \right]$$

Note that Eq. (18) is identical to the previous results^65^, except that *c*_*p*_ is replaced by *c*_*υ*_. If some assumptions, including a linear temperature profile across the film thickness in the phase B region^63–65^, are adopted, then we can obtain^63–65^ $u = (\rho_{{\text{B}}} - \rho_{\infty } )g(\delta x_{2} - x_{2}^{2} /2)/\mu$, $T - T_{\infty } = (T_{s} - T_{\infty } )(1 - x_{2} /\delta )$, $\dot{m}_{{\text{B}}} = \int_{0}^{\delta } {\rho_{{\text{B}}} u{\text{d}}x_{2} } = {{\rho_{{\text{B}}} (\rho_{{\text{B}}} - \rho_{\infty } )g\delta^{3} } \mathord{\left/ {\vphantom {{\rho_{{\text{B}}} (\rho_{{\text{B}}} - \rho_{\infty } )g\delta^{3} } {3\mu }}} \right. \kern-\nulldelimiterspace} {3\mu }}$, and $\delta = \left\{ {4\mu k(T_{s} - T_{\infty } )x_{1} {\text{ }}/\rho _{{\text{B}}} (\rho _{{\text{B}}} - \rho _{\infty } )g\left[ {i_{{{\text{AB}}}} + 3c_{\upsilon } (T_{s} - T_{\infty } )/8} \right]{\text{ }}g\left[ {i_{{{\text{AB}}}} + 3c_{\upsilon } (T_{s} - T_{\infty } )/8} \right]} \right\}^{{1/4}}$, where *g* is the acceleration of gravity; hence *h* = *k*/*δ*. To sum up, the theoretical convective heat transfer coefficients consistently determined by Eqs. (16)–(18) are the functions of velocity (or mass flow rate), temperature difference and fluid properties for both single-phase and phase-change flows, as is absolutely different from the proposed advective constant *α* = *ρcu*_*∞*_. The velocity and temperature expressions in Eqs. (16)–(18) depend on the Navier–Stokes equations, energy equation, and other known conditions. Now, we establish the 3D energy and entropy transfer theory of thermal convection, then the advective constant *α* and the convective heat transfer coefficient *h* are successfully derived from this theory and analytically presented by the representation of *α* = *ρcu*_∞_ and Eqs. (16)–(18) for single-phase and phase-change flows. These expressions and their revealed heat transfer mechanism of convection make the original and modern Newton’s laws of cooling become the complete scientific laws.

## Experiments

### Internal laminar experiment

**Figure 5:**
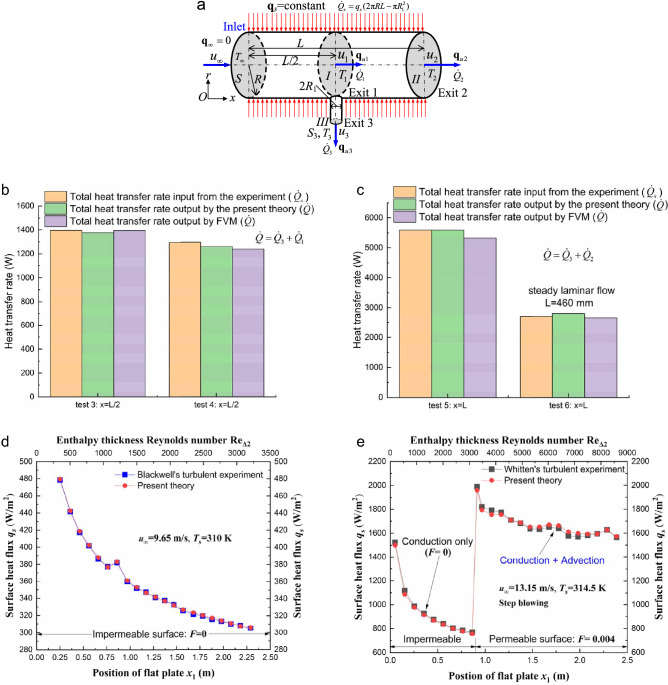
Experimental validations of the 3D convective heat flux theory. (**a**) A steady, incompressible, internal laminar flow with one inlet and two exits (exits 2 and 3) is considered. (**b**) The total heat transfer rates obtained from the theoretical heat flux in Eq. (12) and FLUENT software are compared with those experimentally obtained for the internal laminar flow of water in a half-length (*L*/2) and (**c**) a full-length (*L*) pipe. (**d**) Correlation of the experimental surface heat flux of air obtained by Blackwell^26^ in the turbulent flow over a horizontal, impermeable flat plate with the present analytical distribution determined by Eq. (13). (**e**) Comparison of the surface heat flux of air on the impermeable plus permeable flat plate in the turbulent flow experiment (step blowing) carried out by Whitten^26^ with that from the present convective heat flux theory.

To verify the present convective heat flux theory, a test facility is designed and constructed to investigate the steady laminar flow of incompressible, constant-property water through a circular tube with one inlet and two exits (Fig. 5a). The details of the test rig are presented in reference^25^. Constant heat flux **q**_*s*_ is applied on the circular pipe wall (radius *R*), connected to the small bypass tube exit *III* of radius *R*_1_. The velocity and temperature at surfaces *I* and *II* (or *III*) refer to the mean values across the entire surface, and *S* represents the cross-sectional area. Streamwise conduction is neglected. The rate of conductive heat transfer $\dot{Q}_{s}$ entering across the tubular wall, originally supplied by the constant heating power during the laminar experiment, is compared with the rate of total heat transfer $\dot{Q}$ leaving across sections *III* and *I* (or *II*) $\dot{Q} = \dot{Q}_{3} + \dot{Q}_{1}$ (or $\dot{Q} = \dot{Q}_{3} + \dot{Q}_{2}$), which can be determined by Eq. (12), for the half-length pipe (*x* = *L*/2, Fig. 5b) in tests 3 and 4 or the full-length pipe (*x* = *L*, Fig. 5c) in tests 5 and 6. The rate of total heat transfer $\dot{Q}$ leaving sections *III* and *I* (or *II*) is also numerically calculated by FLUENT software using the finite volume method (FVM)^25^, as indicated in Table 1. Good agreement can be seen between any two of the experimental, numerical and theoretical results^25^, as shown in Fig. 5b,c and Table 1. Note that there are still small differences between the experimental or numerical results and the present theory, one of main reasons is that the average velocity and temperature values across the surfaces *I*, *II* and *III* have to be adopted in Eq. (12) for simplicity, but the experimental or numerical results are obtained from the 2D distributions of velocity and temperature across the surfaces *I*, *II* and *III* satisfying momentum and energy conservations.

**Table 1:** Comparison of the theoretical, experimental and numerical convective heat fluxes.

Test no.	Test 3 (*x* = *L*/2)		Test 4 (*x* = *L*/2)		Test 5 (*x* = *L*)		Test 6 (*x* = *L*)	
Constant surface heating power *P* (W)	1440.45		1359.30		5765.80		2884.90	
Heat loss power *P*_*loss*_ (W)	44.78		63.62		176.34		177.04	
Experimental surface heat transfer rate *P*_*net*_ = *P* − *P*_*loss*_ (W)	1395.67		1295.68		5589.46		2707.86	
Theoretical total heat transfer rate $\dot{Q}$ (W)	351.08 ($\dot{Q}_{3}$)	1025.75 ($\dot{Q}_{1}$)	779.95 ($\dot{Q}_{3}$)	481.96 ($\dot{Q}_{1}$)	4189.05 ($\dot{Q}_{3}$)	1399.71 ($\dot{Q}_{2}$)	1050.88 ($\dot{Q}_{3}$)	1748.84 ($\dot{Q}_{2}$)
	1376.83		1261.91		5588.76		2799.72	
Numerical (FVM) total heat transfer rate $\dot{Q}$ (W)	1394.07		1239.62		5326.75		2654.88	

How the numerical results and the heat loss power (owing to radiation and others) are obtained can be seen in reference^25^.

### External turbulent flow measurements

Comparing the analytical heat flux in a turbulent flow over an impermeable wall or through a porous surface with the benchmark turbulent flow measurements carried out by Blackwell^26^ or Whitten^26^ is of interest. The conditions of an isothermal wall and a zero-pressure gradient are tightly controlled in both experiments^26^, so free-stream velocity *u*_∞_ remains constant (Fig. 4a). The constant wall temperature *T*_*s*_ is controlled at 310 K in Fig. 5d or 314.5 K in Fig. 5e, and the blowing fraction is defined as *F* = *ρu*_*s*_/(*ρ*_∞_*u*_∞_)^26,31,61,62^. Measurement data points, taken from the thermal boundary layer flows on impermeable or permeable flat plates (Fig. 4a) with uniform blowing (blowing fraction *F* > 0) and suction (*F* < 0), are compared with the theoretical solutions from Eq. (13). All the benchmark turbulent flow measurement data are open and from the textbook by Kays and Crawford^26^. As shown in Fig. 5e, for the impermeable wall surface on the left-hand side (*F* = 0), only conduction occurs; for the porous surface of the same plate on the right-handed side (*F* = 0.004), both advection and conduction contribute to the total convective heat transfer through the wall. The agreement between the benchmark experiments and present formulae is extremely good for all suction and blowing values (Fig. 5e) and for those points on the impermeable wall (Fig. 5d). Note that the convective heat flux *q*_*s*_ through porous surfaces combines the contributions of conduction and advection rather than conduction alone, such as for impermeable surfaces. Additionally, unlike the streamwise advective heat flux **q**_*u*_, the wall-normal heat flux due to advection through the porous surface has a magnitude comparable to that due to conduction, which is evident from the step blowing experiment (Fig. 5e).

In conclusion, to make Newton’s cooling law a complete, consistent, scientific law, we theoretically determine analytical expressions for the advective constant and the convective heat transfer coefficients in terms of the convective heat flux vector for external and internal single-phase and phase-change flows. Although advective constant *α* in the original version of Newton’s law of cooling is different from *h* defined by Fourier and firmly established, *α* can be viewed as the particular inviscid fluid case of *h*, and the dimensionless number bridging the two is the Stanton number. A unified 3D energy transfer theory of thermal convection is built in which formulae of the advective heat flux and entropy flux vectors and entropy generation rate within the system are derived for steady, compressible, single-phase and phase-change flows. The energy transfer mechanism of advection is clearly revealed. Heat advection is unambiguously distinguished from other energy transfer interactions, including mass flow and heat conduction. We have theoretically demonstrated that advection can be considered an independent heat transfer mode induced by the *net total energy transfer* between *two surfaces* due to mass flow. Based on this analysis, advection (or convection) can be considered a fundamental heat transfer mode in addition to conduction and radiation. The present convective heat flux theory has been verified by laminar experiments and turbulent flow benchmark measurements for an incompressible fluid, but further experimental investigations on natural convection and phase transitions for compressible flows are still needed to elucidate this complicated convective mechanism. How this conclusion translates to advective heat transfer in unsteady flows is not clear. The heuristic viewpoint that advection is the net total energy transfer via mass flow in a compressible flow and the analytical determination of the convective heat transfer coefficient broaden the fundamental approaches for designing and enhancing (or weakening) convective heat transfer. Moreover, the present 3D formula for the advective heat flux vector has the potential to be considered the linear phenomenological equation of heat advection for analysis of nonequilibrium thermodynamics^51^.

## Data Availability

The data that support the finding of this study are available from the corresponding author upon reasonable request.

## Appendix: Derivation of the advective heat flux in Eq. (5)

$\dot{Q}_{u} = \dot{m}\left[ {(i + e_{m} ) - (i_{\infty } + e_{m\infty } )} \right]$ in Eq. (3) can be recast into the integral form from inlet surface *I* to any surface *II* (Fig. 1e), which gives $\dot{Q}_{u} = \dot{m}\int_{I}^{II} {{\text{(d}}i + {\text{d}}e_{m} )}$. Following the quasi-equilibrium assumption^19,46,52^ without shaft work and viscous dissipation and considering the Bernoulli Eq. ^52,68^ yields ${\text{d}}e_{m} = - \upsilon {\text{d}}p$, so that

19 $$\dot{Q}_{u} = \dot{m}\int_{I}^{II} {{\text{(d}}i - \upsilon {\text{d}}p)}$$

By using the Gibbs equations and the Maxwell relation^27,47^, one obtains

20 $$\left\{ \begin{gathered} {\text{d}}p = \left. {\frac{\partial p}{{\partial T}}} \right|_{\upsilon } {\text{d}}T + \left. {\frac{\partial p}{{\partial \upsilon }}} \right|_{T} {\text{d}}\upsilon = \frac{\beta }{\kappa }{\text{d}}T - \frac{1}{\upsilon \kappa }{\text{d}}\upsilon \, \hfill \\ {\text{d}}\upsilon = \left. {\frac{\partial \upsilon }{{\partial T}}} \right|_{p} {\text{d}}T + \left. {\frac{\partial \upsilon }{{\partial p}}} \right|_{T} {\text{d}}p = \upsilon \beta {\text{d}}T - \upsilon \kappa {\text{d}}p \, \hfill \\ {\text{d}}T = \left. {\frac{\partial T}{{\partial \upsilon }}} \right|_{p} {\text{d}}\upsilon + \left. {\frac{\partial T}{{\partial p}}} \right|_{\upsilon } {\text{d}}p = \frac{1}{\upsilon \beta }{\text{d}}\upsilon + \frac{\kappa }{\beta }{\text{d}}p \hfill \\ \end{gathered} \right.$$

The change in specific enthalpy *i* can be given in terms of independent properties *T* and *p* or *T* and *υ*

21 $${\text{d}}i = \left. {\frac{\partial i}{{\partial T}}} \right|_{p} {\text{d}}T + \left. {\frac{\partial i}{{\partial p}}} \right|_{T} {\text{d}}p,{\text{ d}}i = \left. {\frac{\partial i}{{\partial T}}} \right|_{\upsilon } {\text{d}}T + \left. {\frac{\partial i}{{\partial \upsilon }}} \right|_{T} {\text{d}}\upsilon$$

According to Bridgman’s relations^47^, these first partial derivatives are in the form

$$\left. {\frac{\partial i}{{\partial T}}} \right|_{p} = c_{p} , \, \left. {\frac{\partial i}{{\partial p}}} \right|_{T} = (1 - \beta T)\upsilon , \, \left. {\frac{\partial i}{{\partial T}}} \right|_{\upsilon } = c_{\upsilon } + \frac{\beta \upsilon }{\kappa }\left. {, \, \frac{\partial i}{{\partial \upsilon }}} \right|_{T} = \frac{\beta T - 1}{\kappa }$$

By inserting into Eq. (21) and considering Eq. (20), the integrand in Eq. (19) can be rewritten as

22 $${\text{d}}i - \upsilon {\text{d}}p = c_{p} {\text{d}}T - \beta T\upsilon {\text{d}}p,{\text{ d}}i - \upsilon {\text{d}}p = c_{\upsilon } {\text{d}}T + {{\beta T} \mathord{\left/ {\vphantom {{\beta T} \kappa }} \right. \kern-\nulldelimiterspace} \kappa }{\text{d}}\upsilon$$

By integrating Eq. (22) from the state of *T*_∞_ and *p*_∞_ (*υ*_∞_) at inlet surface *I* to the state of *T* and *p* (*υ*) at some arbitrary surface *II* and considering the inherent relation between $\dot{Q}_{u}$ and the advective heat flux vector **q**_*u*_, Eq. (19) can be rewritten as

23 $$\dot{Q}_{u} = \int_{S} {({\mathbf{q}}_{u} \cdot {\mathbf{n}}){\text{d}}S} = \dot{m}\left( {\int_{{T_{\infty } }}^{T} {c_{p} {\text{d}}T^{\prime} - \int_{{p_{\infty } }}^{p} {\beta T\upsilon {\text{d}}p^{\prime}} } } \right) = \dot{m}\left( {\int_{{T_{\infty } }}^{T} {c_{\upsilon } {\text{d}}T^{\prime} + \int_{{\upsilon_{\infty } }}^{\upsilon } {{{\beta T} \mathord{\left/ {\vphantom {{\beta T} \kappa }} \right. \kern-\nulldelimiterspace} \kappa }{\text{d}}\upsilon^{\prime}} } } \right)$$

where *S* is the cross-sectional area of surface *II*; **n** is the unit vector pointing outward, normal to the surface (Fig. 1e); and $\dot{m} = \int_{S} {\rho {\mathbf{U}} \cdot {\mathbf{n}}} {\text{d}}S$ is the mass flow rate (kg/s), by definiton^46,48^. Note that the differential mass *m* (Fig. 1b–d) is so small that it is considered to possess uniform properties^16,46^. Therefore, the bracket terms on the right-hand side of Eq. (23) may be directly combined with the integrand of $\dot{m} = \int_{S} {\rho {\mathbf{U}} \cdot {\mathbf{n}}} {\text{d}}S$; when ∆*S* → 0, dropping the signs for integrals on both sides gives Eq. (5).

## Abbreviations

*a*: Molecular thermal diffusivity of the fluid (= *k*/(*ρc*)), m^2^/s
*A*: Surface area of the body, m^2^
*c*: Specific heat capacity for an incompressible fluid (= *c*_*p*_ ≈ *c*_*υ*_), J/(kg K)
*c*_*p*_: Specific heat capacity at constant pressure, J/(kg K)
*c*_*υ*_: Specific heat capacity at constant specific volume, J/(kg K)
*C*: Heat capacity, J/K
*C*_*T*_ = d*i*/d*υ*: Heat capacity at constant temperature in the first-order phase-change process, Pa
*e*: Specific internal energy, J/kg
*e*_*m*_: Specific mechanical energy, J/kg
*e*_*t*_ = *e* + *pυ* + *e*_*m*_ = *i* + *e*_*m*_: Specific total energy for a flowing fluid, J/kg
$\dot{E}_{g}$: Thermal energy generation rate inside the system, W
*F* = *ρu*_*s*_/(*ρ*_∞_*u*_∞_): Blowing fraction
*g*: Acceleration of gravity (= 9.81 m/s^2^)
*h*: Convective heat transfer coefficient, W/(m^2^ K)
*i* = *e* + *pυ*: Specific enthalpy, J/kg
*i*_AB_ = *i*_B_ − *i*_A_: Specific latent heat of phase change (or specific enthalpy difference between phase B and A fluids), J/kg
**J**_*s*_: Entropy flux vector W/(m^2^ K)
*k*: Thermal conductivity of the fluid, W/(m K)
*L*: Total length of the experimental circular tube, m
$\dot{m} = \int_{S} {\rho {\mathbf{U}} \cdot {\mathbf{n}}} {\text{d}}S$: Mass flow rate, kg/s
**n**: Unit vector normal to surface
*p*: Pressure, Pa
*p*_AB_: Constant saturation pressure from phase A to phase B, Pa
*P*: Constant surface heating power applied in the laminar experiment, W
*P*_*loss*_: Heat loss power, W
*P*_*net*_ = *P* − *P*_*loss*_: Net input of conductive heat transfer rate through the wall surface, W
*q*_*j*_: Total convective heat flux component (*j* = 1, 2 and 3), W/m^2^
${\mathbf{q}} = \left\{ {q_{1} ,q_{2} ,q_{3} } \right\} = {\mathbf{q}}_{k} + {\mathbf{q}}_{u}$: Total (resultant) convective heat flux vector, W/m^2^
$\dot{Q} = \int_{S} {({\mathbf{q}} \cdot {\mathbf{n}}){\text{d}}S}$: Heat transfer rate, W
$\dot{Q}_{u} = \int_{S} {({\mathbf{q}}_{u} \cdot {\mathbf{n}}){\text{d}}S}$: Advective heat transfer rate leaving across arbitrary surface, W
*R*(*R*_1_): Radius of a circular pipe (small bypass tube), m
Re_Δ2_ = *u*_∞_Δ_2_/*ν*: Enthalpy thickness Reynolds number
*s*: Specific entropy transfer, J/(kg K)
*S*: Cross sectional area of inner surface within a fluid stream, m^2^
$\dot{S}_{g}$: Entropy generation rate inside the system, W/K
*T*: Temperature of fluid, K
*T*_*ad*_: Potential temperature (${\text{d}}T_{ad,p}^{{}} = \frac{{\beta T_{ad,p}^{{}} }}{{\rho c_{p} }}{\text{d}}p,{\text{ d}}T_{ad,\upsilon }^{{}} = - \frac{{\beta T_{ad,\upsilon }^{{}} }}{{\kappa c_{\upsilon } }}{\text{d}}\upsilon$), K
*T*_AB_: Constant phase-change temperature or saturation temperature (= *T*_∞_), K
*T*_*s*_: Body’s uniform temperature at wall surfaces, K
*u*_*j*_: Fluid velocity component (*j* = 1, 2 and 3), m/s
*u*_∞_: Inlet velocity for internal flows or free-stream velocity for external flows, m/s
${\mathbf{U}} = \left\{ {u_{1} ,u_{2} ,u_{3} } \right\}$: Fluid velocity vector, m/s
*υ*≡1/*ρ*: Specific volume, m^3^/kg
*V*: Body volume, m^3^
$x = \dot{m}_{B} /(\dot{m}_{A} + \dot{m}_{B} )$: Mass fraction of phase B or quality (= (*υ* − *υ*_A_)/(*υ*_B_ − *υ*_A_) = (*s* − *s*_A_)/(*s*_B_ − *s*_A_))
*x*_*j*_: Space coordinate in the *i* direction in Cartesian system (*j* = 1, 2 and 3)

## Greek symbols

*α*: Proportional coefficient or advective constant in Newton’s original rate equation (= *ρcu*_∞_), W/(m^2^ K)
*β*: Volumetric coefficient of thermal expansion, 1/K
*θ* = *T* − *T*_∞_: Temperature difference, K
*θ*_*s*_ = *T*_*s*_ − *T*_∞_: Wall surface temperature difference, K
*τ*: Time, s
*δ*: Thickness of the boundary-layer or the liquid film, m
*δV*: Incremental volume, m^3^
*ρ*≡1/*υ*: Density of fluid, kg/m^3^
*μ*: Dynamic viscosity of phase B, Pa s
*ν*: Kinematic viscosity (= *μ*/*ρ*), m^2^/s
*κ*: Isothermal compressibility, 1/Pa
Δ_2_: Enthalpy thickness of a thermal boundary layer, m
∇: Gradient sign

## Superscripts

*sen*: Sensible energy transfer
*l**at*: Latent energy transfer

## Subscripts

A(B): Phase A (phase B)
AB: Phase transition process from phase A to phase B
*ad*: Potential temperature (or adiabatic temperature) condition
*av*: Average value across the cross-sectional area of fluid in an internal flow (*T*_*av*_) or across the wall surface (*h*_*av*_)
*b*: Body considered in the original Newton’s law of cooling
*g*: Generation of thermal energy (or entropy)
*j*: *j* = 1, 2 And 3 represent the streamwise, wall-normal and transverse directions in Cartesian system, respectively
*k*: Conduction condition in convective heat transfer
*m*: Mechanical energy
*p*: Potential temperature expressed by the variable of pressure
*r*, *φ*, *x*: Radial, circumferential and axial directions in cylindrical system, respectively
*s*: Fluid condition at the wall surface
*t*: Total energy
*u*: Advection condition in convective heat transfer
*υ*: Potential temperature expressed by the variable of specific volume
∞: Constant fluid condition at the inlet for internal flows or free-stream condition for external flows
